# Pediatric Solid Tumors in Resource-Constrained Settings: A Review of Available Evidence on Management, Outcomes, and Barriers to Care

**DOI:** 10.3390/children5110143

**Published:** 2018-10-23

**Authors:** Nicholas H. Carter, Andrew H. Avery, Jaime Libes, Harold N. Lovvorn, Erik N. Hansen

**Affiliations:** 1 Department of Surgery, Vanderbilt University Medical Center, Nashville, TN 37232, USA; andrew.avery@vumc.org; 2 Department of Pediatrics, University of Illinois College of Medicine at Peoria, Peoria, IL 61605, USA; libesjm@uic.edu; 3 Department of Pediatric Surgery, Vanderbilt University Medical Center, Nashville, TN 37232, USA; harold.lovvorn@vumc.org (H.N.L.III); erik.n.hansen@vumc.org (E.N.H.); 4 Department of Paediatric Surgery, AIC-Kijabe Hospital, Kijabe 00220, Kenya

**Keywords:** Wilms Tumor, pediatric solid tumor, low and middle-income countries (LMICs), disparities, barriers to care

## Abstract

International disparities in outcomes from pediatric solid tumors remain striking. Herein, we review the current literature regarding management, outcomes, and barriers to care for pediatric solid tumors in low- and middle-income countries (LMICs). In sub-Saharan Africa, Wilms Tumor represents the most commonly encountered solid tumor of childhood and has been the primary target of recent efforts to improve outcomes in low-resource settings. Aggressive and treatment-resistant tumor biology may play a role in poor outcomes within certain populations, but socioeconomic barriers remain the principal drivers of preventable mortality. Management protocols that include measures to address socioeconomic barriers have demonstrated early success in reducing abandonment of therapy. Further work is required to improve infrastructure and general pediatric care to address disparities.

## 1. Introduction

### 1.1. Rationale

Disparities in outcomes from pediatric solid tumors remain striking. In high-income countries (HICs), several multi-institutional collaborations, including the International Society of Pediatric Oncology (SIOP) and the National Wilms Tumor Study Group (NWTSG; now, the Children’s Oncology Group, COG) have succeeded in raising overall survival for Wilms Tumor (WT) to greater than 90% at five years [1,2,3]. In contrast, children with most cancers, and especially WT and other solid malignancies, residing in low- and middle-income countries (LMICs) face persistently high mortality rates [4]. Recent years have seen increased efforts to improve these alarmingly poor outcomes [5].

### 1.2. Objectives

This review seeks to report available literature regarding the burden of disease, management, and outcomes from pediatric solid cancers in low-resource settings, as well as published evidence regarding barriers to care.

## 2. Materials and Methods

Medline and Cochrane database searches were performed in July 2018. Search terms included “Wilms tumor, Africa”, and “pediatric tumor, Africa.” The reference lists of articles identified using these search terms were also reviewed. Studies were selected based on the relevance to burden of disease, management, and outcomes of pediatric solid tumors in LMICs.

## 3. Results

### 3.1. Nephroblastoma (Wilms Tumor)

#### 3.1.1. Burden of Disease and Current Outcomes

Most published literature on the burden of disease and outcomes from pediatric solid tumors in LMICs were derived from sub-Saharan Africa (SSA) where disparities are alarming. In a recent review of cancer registries in selected major urban centers in Zimbabwe, Uganda, and Kenya, WT was the most common solid tumor identified in children and third most common pediatric cancer after leukemia and lymphoma. Survival at one year was only 61%, 39%, and 66% at each respective site [4]. Five-year survival was 33% and 7.9%, with the rate at the third site unavailable due to all children being lost to follow-up. In other recent studies from Kenya, two-year survival has ranged between 35% and 52% [6,7]. There are considerable intra-country and inter-country disparities in access to care and health expenditures, but outcomes data from remote and particularly low-resource settings are limited. At a center in Malawi with self-reported limited resources, projected survival with a median follow-up of 16 months was similarly poor at 46% [8]. The lowest reported survival was 11% from a center with extremely low rates of completion of therapy during a period impacted by armed conflict [9].

#### 3.1.2. Biology

Hereditary predisposition and biologic behavior of tumors may contribute to disparate outcomes across ethnic groups. Over-representation of certain Kenyan tribes among patients treated for WT suggests increased incidence within specific populations [10]. Using an unbiased proteomic screen to explore biologic variables, peptide profiles of WT originating in Kenyan patients differed considerably from signatures found in both black and white patients residing in North America [11]. Relative to North American controls, the molecular signatures of Kenyan WTs expressed markers of adverse behavior and treatment resistance [12,13]. The relative impact of these biologic differences on survival is difficult to characterize in low-resource settings where access to care is limited, and the impact of late presentation on favorable outcomes is yet to be determined. Members of our research group also have demonstrated the feasibility and utility of molecular characterization of WT specimens in Baghdad, Iraq, as a paradigm for specimen collection, evaluation, and assessment of treatment implication in other LMICs [14].

#### 3.1.3. Barriers to Care

Inadequate access to and abandonment from care remain the likely principal drivers of mortality [15]. Efforts to identify and treat children with WT remain hampered by the “Three Delays”: Deferred presentation, late diagnosis, and inadequate treatment [16]. Late presentation of WT is commonly observed in LMICs and has a clear association with poor outcomes. In a review of 150 consecutive patients treated at a major referral center in South Africa, only 6% of WT patients presented with Stage I disease, while 65% presented with Stage III or IV disease, which is in stark contrast to Stage distribution in North America and Europe [17]. Patients presenting with early-stage disease had nearly 90% survival, while those with late-stage presentation faced considerably lower survival rates [18]. Moreover, WT that present with advanced stage disease may have progressed further along the pathogenic sequence and have acquired treatment resistant biologic features, such as *TP53* mutation and *MYCN* alteration [13]. Accurate diagnosis and staging of WT may be delayed in some settings, given limited access to pediatricians who have oncologic training, to pathologists with experience in childhood cancers, and to skilled sonographers [19].

Completion of therapy for WT in LMICs remains a significant challenge. A review of outcomes from eight referral centers in SSA found treatment abandonment rates ranging from 14–48% [20]. Root causes for abandonment of therapy are complex and include socioeconomic pressures, geographic barriers, disparities in health literacy, and cultural biases [21,22]. Inability to complete therapy on an outpatient basis has led clinicians in some settings to take the dramatic step of admitting children from remote provinces for the entire duration of their chemotherapy [23]. Fear of hospital detention may contribute to delayed presentation and/or abandonment of therapy [24]. Drug shortages and counterfeit production can severely restrict local availability to proper chemotherapeutic agents [25]. Human resources remain a limiting factor in many settings with inaccess to pediatric surgeons, oncologists, and nursing staff with pediatric oncology qualifications. Co-existent human immunodeficiency virus (HIV) infection and/or severe malnutrition may complicate efforts to initiate and complete therapy. In regions with a high HIV burden, up to 20% of pediatric patients have lost a parent, thus impacting family support and potentially requiring additional intervention to carry a child to completion of therapy [26]. Post-treatment surveillance of pediatric patients with WT is also difficult; the referral centers in Zimbabwe, Uganda, and Kenya reported lost-to-follow-up rates of 15–43% in the first year after treatment [4].

Barriers to care can be reduced through social programs. Excellent evidence exists that health insurance reduces mortality for children burdened with cancer in LMICs. For example, in Kenya, children with WT who were enrolled in the National Hospital Insurance Fund were more likely to initiate therapy, complete preoperative chemotherapy, undergo operative resection, complete post-operative chemotherapy, and finish radiation therapy [7]. Health insurance is especially important for providing equity in access to early diagnosis [27]. Although not formally evaluated to date, our experience suggests that efforts to improve family education and health literacy regarding these challenging and difficult to treat cancers provide an additional means to retain children in therapy until completion.

#### 3.1.4. Management

Two strategies for the multidisciplinary treatment of WT have been employed yielding comparable results in HICs. The COG in North America practices operative resection of renal tumors followed by adjuvant chemotherapy for certain risk groups. In Europe, the SIOP initiates preoperative chemotherapy of all renal tumors without biopsy, which potentially can downstage the tumor at surgery and reduce tumor rupture, intensity of postoperative chemotherapy, and need for radiotherapy [28]. Both approaches have been extrapolated to LMICs, although currently no long-term outcome data are available to compare efficacy and feasibility of either approach in low-resource settings. Each approach has potential advantages in LMICs: The COG strategy allows earlier resection and confirms diagnosis before initiation of precious treatment with often scarce medications. In contrast, the SIOP approach may better address late presentation of large tumors in settings of limited or non-existent access to radiotherapy [29]. In low-resource settings, therefore, a hybrid approach taking advantage of both strategies may be best suited to optimize chances of cure of the child presenting with a renal tumor. For example, the United Kingdom Children’s Cancer Study Group, which utilizes initial biopsies for diagnosis verification before implementing neoadjuvant treatment and histology for prognosis, may prove to be a useful middle road in LMIC settings, though some controversy exists regarding the significance of biopsy-tract tumor seeding [30]. The most updated SIOP recommendations allow for biopsy when a WT diagnosis is in question following imaging analysis and, further, recommend initial nephrectomy in very young patients who are of greater likelihood to have other renal tumor types, such as congenital mesoblastic nephroma, thereby minimizing risk of over- or mis-treatment of non-WT tumors [31].

Recent years have seen renewed efforts to standardize treatment protocols for management of WT in LMICs. The SIOP Paediatric Oncology in Developing Countries (PODC) committee published clinical guidelines for management and supportive care of WT patients in low-income settings, which outline the requirements for therapy with curative intent [19,32]. These guidelines offer specific recommendations for addressing socioeconomic and health literacy factors that contribute to abandonment of care, such as providing free treatment and lodging for poor families and counseling of guardians regarding WT and the importance of completing therapy. In a prospective trial spanning eight referral centers in five SSA nations, implementation of these guidelines accomplished increased rates of patients alive without evidence of disease at the end of treatment and decreased abandonment of treatment [33].

### 3.2. Other Solid Tumors of Childhood

WT represents the most common and intervenable solid tumor of childhood and so has been the target of the greatest efforts to improve outcomes in LMICs given its profound disparity with industrialized countries. Socioeconomic factors among patients and families that limit WT treatment completion are likely similar among other pediatric solid tumor patients. SIOP-PODC guidelines for management of neuroblastoma and retinoblastoma in LMICs are now also available [34,35]. A review of outcomes for neuroblastoma at a center in South Africa found that nearly 75% of patients presented with metastatic disease. Three-year survival was only 4% [36]. The reported incidence of neuroblastoma in LMICs appears to be lower than in HICs, perhaps due to unique environmental exposures, different genetic predisposition or vulnerabilities, under-diagnosis, or delayed presentation with advanced, non-treatable disease [37]. Biological data for neuroblastoma are largely unavailable from LMICs, and the limited capacity for biological tumor characterization in LMIC treatment centers inhibits tumor risk stratification, which together may lead to overtreatment, contributing to incentives for treatment abandonment [34]. Retinoblastoma has a higher prevalence in LMICs of SSA compared to HICs, and confers a similar disparity profile to WT and neuroblastoma, with a lower survival and higher rate of late-stage presentation. Less availability of disease-specific chemotherapy and ancillary oncologic services is thought to drive this poorer outcome among LMICs [38]. Liver tumors, including hepatoblastoma and hepatocellular carcinoma, may be more common in LMICs than in HICs and carry a devastatingly poor prognosis [39,40]. Aside from socioeconomic barriers to completing treatment, personnel, expertise, and infrastructural limitations at the level of treatment centers have been demonstrated to independently contribute to worse outcomes for pediatric sarcoma patients in middle-income countries [41]. Thus, in addition to assessing and overcoming patient and family barriers to solid tumor care, there is a need for an emphasis on improving resource availability and treatment consistency across treatment centers in LMICs.

## 4. Discussion

Recent literature indicates that mortality rates for pediatric solid tumors in LMICs remain unacceptably high due to complex interactions between social and economic status, access to care, race and ethnicity, health literacy, and tumor biology. Collectively, late presentation with high risk for treatment abandonment, on-therapy mortality due to deficiencies in supportive care, and aggressive and treatment-resistant biology remain the biggest factors driving mortality in poor settings. Despite persistent challenges, there are great gains to be made in this field. When working with a young population of patients, many of whom have eminently treatable disease, the impact of systemic improvements in care in terms of disability-adjusted life years and quality-adjusted life years may be profound [42].

Since socioeconomic barriers are major obstacles to successfully completing therapy for pediatric solid tumors in LMICs, we are encouraged to see improved outcomes when these obstacles are specifically addressed. The success of eight SSA centers to reduce abandonment of treatment through implementation of PODC guidelines, including financial and social support for poor families, represents a key step towards reducing widespread disparities. Emphasis and assistance for families to enroll in national health insurance plans has proven benefits on survival at least from WT. However, much work remains to be completed in expanding access to this type of support and providing scalable, sustained, multi-disciplinary prevention of treatment abandonment.

To overcome this burden and major cancer health disparity in LMIC settings, our group has emphasized the professional training of native, in-country pediatric surgeons, anesthesiologists and anesthetists, oncologists, intensivists, radiologists, and pathologists. Indeed, we have begun to witness a growing number of these health care providers assume increasing responsibility and independence in the care of the often-complex pediatric cancer patients (See Appendix A). Moreover, personal communication with numerous local physicians in these low-resource environments, such as Kenya, Uganda, and Iraq, has confirmed a keen interest to learn and acquire improved and new research techniques, both bed side and bench top, that will surely lead to improved outcomes long term. These interests may be further supported by an expansion of international partnerships and academic twinning.

Attempts to clarify population-specific tumor biology have revealed unique molecular profiles that, with additional work, could yield novel druggable targets and optimized, more precise care schemes in low-resource settings. Recent research suggests that population-specific genetic variations may increase risk for adverse drug reactions to commonly-prescribed antineoplastic agents, and contribute to difficulty completing therapy in particular settings [43]. Programs to enhance family understanding of the disease and treatment plan (i.e., health literacy) should help to retain children in care and improve outcomes also.

Limitations of this review include the heterogeneous nature of populations within the referenced studies. It is important to note the vast diversity in socioeconomic and medical environments included within the umbrella terms of “LMICs” or “low-resource settings.” Much of the available literature stems from referral centers in urban settings in SSA. The full extent of geographic obstacles to care felt by rural populations may be poorly represented. This review focuses on WT as a pediatric solid tumor that has been proven in HICs and even in certain LMIC settings to be highly intervenable. The available evidence suggests that overall outcomes for other solid tumors of childhood in LMICs remain dismal.

Efforts to improve oncologic outcomes in LMICs may be hampered by competing health priorities. Prior authors have noted the need for interventions that address oncologic disparities while strengthening infrastructure for general pediatric care [44,45]. As one group stated succinctly: “Sharpening the needlepoint of surgical expertise will, of itself, not compensate for the major infrastructural deficiencies, but must proceed in tandem with resource development and allow health planners to realize that pediatric surgical oncology is a cost-effective service than can uplift regional services” [46]. This tandem development of infrastructure and therapeutic interventions remains the central challenge to addressing disparities in outcomes for poor children in low-resource settings. We hope that this review will help amplify the chorus of calls to address the alarming disparity in outcomes from specific solid tumors arising in children across the globe.

## Figures and Tables

**Figure A1:**
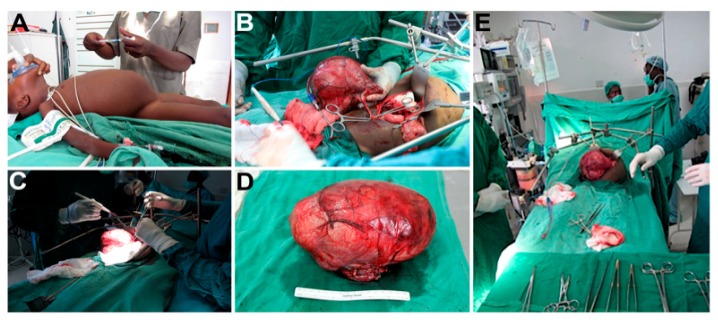
(**A**) Preoperative abdominal mass in a toddler; (**B**) isolation of Wilms Tumor; (**C**) dissection of Wilms Tumor; (**D**) surgical specimen; (**E**) intraoperative abdominal mass in a toddler.
